# Non Breast-Milk-Fed Very Preterm Infants Are at Increased Risk of Iron Deficiency at 4–6-Months Corrected Age: A Retrospective Population-Based Cohort Study

**DOI:** 10.3390/nu16030407

**Published:** 2024-01-30

**Authors:** Grace Power, Lisa Morrison, Ketan Kulkarni, Hudson Barr, Marsha Campbell-Yeo, Balpreet Singh, Alexandra Stratas, Carmen Landry, Michelle Higgins, Satvinder Ghotra

**Affiliations:** 1 IWK Health Centre, Halifax, NS B3K 6R8, Canada; 2 Faculty of Medicine, Dalhousie University, Halifax, NS B3H 4R2, Canada

**Keywords:** iron deficiency, preterm, formula-fed, breast-fed

## Abstract

Iron supplementation is routinely recommended for breast-milk-fed preterm infants. However, the Canadian Pediatric Society recommends no additional iron supplementation for preterm infants fed primarily with iron-rich formula. Other pediatric societies don’t provide specific guidance on supplemental iron for formula-fed preterm infants. This study investigated how feeding type influences iron status of very preterm infants at 4–6-months corrected age (CA). A retrospective cohort study was conducted using a population-based database on all very preterm infants (<31 weeks gestational age) born in Nova Scotia, Canada from 2005–2018. Information about feeding type, iron intake from formula, supplemental iron therapy and iron status at 4–6-months CA was extracted. Iron deficiency (ID) was defined as serum ferritin <20 and <12 µg/L at 4-and 6-months CA, respectively. Of 392 infants, 107 were “breast-milk-fed” (exclusively or partially) and 285 were “not breast-milk-fed” (exclusively fed with iron-rich formula) at 4–6-months CA. Total daily iron intake was higher in the non-breast-milk-fed group (2.6 mg/kg/day versus 2.0 mg/kg/day). Despite this, 36.8% of non-breast-milk-fed infants developed ID versus 20.6% of breast-milk-fed infants. ID is significantly more prevalent in non-breast-milk-fed infants than breast-milk-fed infants despite higher iron intake. This suggests the need to revisit recommendations for iron supplementation in non-breast-milk-fed preterm infants.

## 1. Introduction

Iron deficiency (ID) during infancy, regardless of concurrent anemia, is associated with poor motor, cognitive, socioemotional, and behavioral outcomes later in life [1,2,3,4]. In children under the age of 2, ID may have irreversible effects on the developing brain, irrespective of subsequent iron therapy [2,5]. Preterm infants are considered at increased risk for developing ID [2,6,7,8]. Thus, it is a universal practice to provide prophylactic iron supplementation to these infants [8,9,10]. Despite this, some preterm infants develop ID [11,12]. In a recent study, 32% of very preterm infants (VPI, <31 weeks’ gestational age at birth) developed ID by 6-months of age, despite prophylactic iron supplementation [12].

Breast-milk-fed preterm infants are considered to be at particularly increased risk for ID due to breast-milk’s low iron content [13]. Therefore, iron supplementation is routinely recommended for these infants [14,15,16]. However, preterm infants fed iron-rich formula are generally deemed to be at lower risk, as these formulas contain more iron. The Canadian Pediatric Society (CPS) currently recommends no additional iron supplementation for preterm infants weighing <2 kg at birth if they are primarily fed with iron-rich formula (>10 mg/L of iron) [14]. The American Academy of Pediatrics (AAP) and European Society of Pediatric Gastroenterology, Hepatology and Nutrition (ESPGHAN) make general recommendations about the iron requirements of preterm infants, which may range from 2–4 or 2–3 mg/kg/day, but do not provide specific guidance on iron requirements of preterm infants fed with iron-rich formula versus those who are breast-milk-fed [15,16].

There is a dearth of literature examining iron status in VPI fed with breast-milk versus those fed with formula. Recent work by our group demonstrated that formula feeding at 4–6 months CA is an independent risk factor for ID [12]. Infants who were exclusively formula fed at 4–6 months CA had higher odds of developing ID compared to infants who were exclusively or partially breast-milk fed. It was not clear from this study though if this association was observed due to lower iron intake in the formula feeding group or due to decreased bioavailability of iron from the formula [12]. The current study investigated this relationship in detail by collecting information on iron intake in the whole study cohort. Thus, the primary objective of this study was to explore the relationship between feeding type and ID in VPI. A secondary objective was to explore risk factors associated with ID in exclusively formula-fed VPI. 

## 2. Materials and Methods

This retrospective population-based cohort study was conducted at IWK Health using the population-based AC Allen Perinatal Follow-up Program (PFUP) database. Each VPI born in the province of Nova Scotia, Canada is enrolled in the PFUP database, which prospectively captures data concerning their perinatal course, neonatal course, and follow-up. This study included all VPI born in Nova Scotia between 2005 and 2018. Exclusions included infants with congenital malformations, chromosomal anomalies, or hemolytic anemias or those fed with cow’s milk or non-iron rich formula containing <10 mg/L of iron. Infants residing outside the Halifax area were followed through travel clinics for geographical reasons and also excluded, since blood testing could not be performed in follow-up for these patients. 

All eligible VPI were fed as per the IWK NICU feeding guidelines. Prophylactic iron supplements (2–3 mg/kg/day) in the form of ferrous sulphate were started at 2–4 weeks chronological age in accordance with the CPS guidelines [17]. Infants born ≥1000 g were started on 2–3 mg/kg/day of iron, while infants born <1000 g received 3–4 mg/kg/day of iron. For formula-fed infants, iron obtained from formula was taken into consideration while determining the supplemental iron dosage. Further, hemoglobin and serum ferritin levels were regularly measured every 3–4 weeks during the neonatal stay, and iron dosage was adjusted accordingly up to a maximum of 6 mg/kg/day. Erythropoietic stimulants were not used at the study centre. Iron therapy was suspended for 2–3 weeks following any blood transfusion [7]. Continuing iron prophylaxis was recommended at discharge until 9–12-months CA. Each infant was seen through the PFUP for a growth and neurodevelopment check at 4–6-months CA when serum hemoglobin and ferritin levels were again measured (this blood testing could not be performed for infants seen through travel clinics). At this visit, the feeding history (whether the infant was being fed with breast-milk or formula), average daily formula volume intake, and formula brand used) was also collected from the parents. 

This study was approved by the IWK Research Ethics Board (1026862).

All relevant clinical data was extracted from the PFUP database. Electronic health records and scanned copies of medical charts at 4–6-months clinic visit were also accessed to retrieve information on feeding and iron intake. The feeding details were retrieved. Information on iron supplementation and dosage at 4–6-months CA was also extracted. 

A market survey on all formula brands used by study participants was conducted to determine the iron composition of each formula. Iron intake from formula (mg/kg/day) was then calculated for each infant by multiplying the iron content of the formula (mg/mL) by average daily formula volume intake and dividing by the infant’s weight. Iron obtained from supplements (mg/kg/day) was calculated for each infant by dividing daily iron supplementation dose (mg/day) by the infant’s weight. Each infant’s total daily iron intake was then calculated by summing iron obtained from formula and supplements. ID was defined as serum ferritin <20 µg/L or <12 µg/L at 4- and 6-months respectively. Iron overload was defined as ferritin >300 µg/L [18]

Other variables of interest included antenatal variables (maternal age, maternal anemia, gestational hypertension, smoking, maternal diabetes, antepartum hemorrhage, multiple birth, and mode of delivery), neonatal variables (gestational age at birth, birthweight, length of hospital stay, need for blood transfusions, hemoglobin at discharge, ferritin at discharge, iron supplementation dose at discharge, sex, bronchopulmonary dysplasia requiring oxygen at 36 weeks, hemodynamically significant patent ductus arteriosus, necrotizing enterocolitis, culture positive sepsis, any grade of intraventricular hemorrhage, and cystic brain injury and post discharge variables at 4–6-month CA (anti-reflux medication use). Information about parental marital status, and urban vs. rural dweller status was also extracted. 

Based on their feeding type at 4–6 months CA, the infants were divided into two groups: breast-milk-fed and non-breast-milk-fed. The non-breast-milk-fed group included infants who were fed with formula alone at 4–6-months CA. Breast-milk-fed infants were exclusively or partially fed with breast-milk at this age. Infants fed partially with breast-milk received some formula, but the proportion of breast- milk versus formula could not be estimated since it was not possible to quantify the intake for babies being directly breast-milk-fed. The two groups were compared. The exclusively formula-fed infants were further divided into those with ID and those without ID.

The breast-milk-fed and non-breast-milk-fed groups were compared using the following analysis process, as were the ID and non-ID formula-fed groups. Continuous variables were listed as means and standard deviations, while categorical variables were listed as percentages. Unpaired t-tests and Chi square or Fisher’s exact tests were used to compare the groups. The results are presented as odds ratios (OR) with 95% CIs for categorical outcomes and mean and standard deviations (SD) for continuous outcomes. Median and interquartile range were also calculated for serum ferritin concentration, which did not follow a normal distribution, and the *p*-value was obtained using the Wilcoxon-Mann-Whitney test. To identify if feeding type at 4–6 months was independently predictive of ID, a multivariable logistic regression analysis was performed to adjust for maternal and neonatal differences between the two groups. To predict ID in formula-fed infants, univariate analyses were performed using logistic regression models. Factors with a *p* value < 0.20 in the univariate analyses were entered into a multivariable risk model for the occurrence of ID using a backward selection procedure. Variables with a *p* value < 0.05 were retained. Statistical significance was defined as *p* < 0.05 with two-tailed tests. Analysis was completed using SPSS Statistics Version 27.

## 3. Results

**Figure 1:**
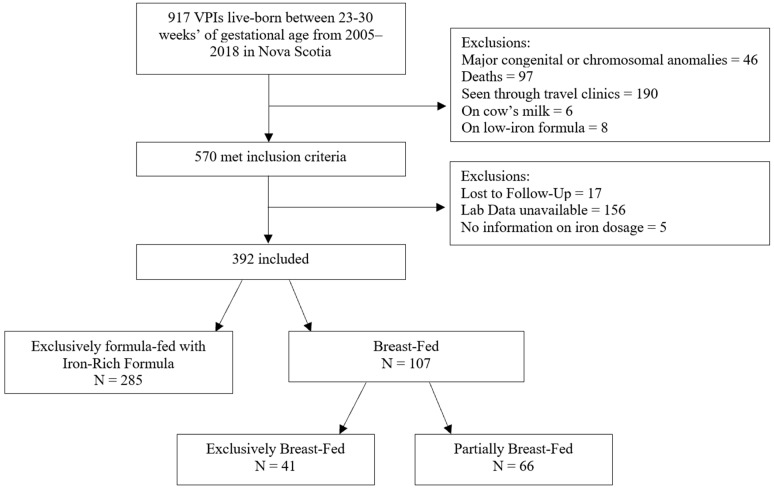
Flow chart of study participants.

Among 917 VPI born live between 23- and 30^6/7^-weeks’ gestation in Nova Scotia from 2005–2018, 570 met study inclusion criteria (Figure 1). Of these, only 17 infants were lost to follow-up and 5 were missing details of iron dosage. Laboratory data was unavailable in 156 infants for various reasons: 86 infants had a clotted sample or no blood testing performed, 64 with insufficient samples for measuring serum ferritin, and 6 patients had a blood testing performed outside the study window. A total of 392 infants were included. Of these, 285 were exclusively fed with iron-rich formula and 107 were breast-milk-fed. Of those in the breast-milk-fed group, 41 were exclusively fed with breast-milk and 66 were partially fed with breast-milk.

Table 1 compares the clinical characteristics of breast-milk-fed and non-breast-milk-fed infants during the NICU stay. The non-breast-milk-fed group had lower mean maternal age and higher prevalence of smoking. Mean dose of supplemental iron at discharge was lower in the non-breast-milk-fed group, and a larger percentage of non-breast-milk-fed infants had single parents. The two groups had no significant differences with respect to gestational age, birth weight or short-term neonatal morbidities. Mean ferritin at discharge was comparable between the two groups.

The clinical characteristics of breast-milk-fed and non-breast-milk-fed infants at 4–6-months CA are depicted in Table 2. Non-breast-milk-fed infants had higher mean daily formula intake and obtained more iron from formula on average than breast-milk-fed infants. In contrast, a greater percentage of breast-milk-fed infants were taking iron supplements (79.4% vs. 57.9%), and they received more iron from supplements on average. The non-breast-milk-fed infants had greater iron intake overall combined from formula and supplements (2.6 mg/kg/day vs. 2.0 mg/kg/day in the breast-milk-fed group). Despite this, a greater percentage of non-breast-milk-fed infants were ID (36.8% vs. 20.6%; OR: 2.25). There were no significant differences in the use of anti-reflux medication between the two groups. Non-breast-milk-fed babies remained at high risk of ID after adjusting for maternal and neonatal variables (OR: 2.11 [1.24, 3.59]; *p* = 0.01). None of the study patients had iron overload. 

Mean iron intake from formula alone in the non-breast-milk-fed group was 1.66 mg/kg/day (Table 2). When calculated from the formula alone (without supplements), the iron intake was >2 mg/kg/day in only 20% of infants.

The iron indices in 2 groups are presented in Table 3. 

Of 285 infants exclusively formula-fed with an iron-rich formula, 105 were found to have ID at 4–6-months CA. Table 4 summarizes their neonatal characteristics. Gestational hypertension was more common in the ID group. ID infants also had a lower mean gestational age, lower mean birth weight, and were more likely to have been born <1100 g. A cut-off of 1100 g was chosen instead of the traditional cut-off of 1000 g because the ID group’s average birthweight was 1069 g compared to 1170 g in the non-ID group. ID was more common in infants who received blood transfusion during their neonatal stay. Other antenatal, neonatal and sociodemographic variables were not significantly different between the 2 groups.

Table 5 compares clinical characteristics of ID and non-ID formula-fed infants at 4–6-months CA. The ID group had significantly lower CA at time of assessment. The proportion of infants on preterm formula post-discharge was higher in the ID group. ID infants had higher daily iron intake from formula and total daily iron intake combined from formula and supplements. There was no significant difference in the use of anti-reflux medication between the 2 groups. 

Other iron indices are presented in Table 6.

Independent predictors of ID in formula-fed VPI included gestational hypertension (OR = 2.03 (1.09, 3.79); *p* = 0.03) and birth weight <1100 g (OR= 1.69 (1.03, 2.77); *p* = 0.04).

## 4. Discussion

This retrospective population-based cohort study investigated the effect of feeding type on iron status of VPI at 4–6-months CA. Authors reported that non-breast-milk-fed infants received more iron on average each day than breast-milk-fed infants. Despite this, non-breast-milk-fed VPI had a higher rate of ID than their breast-milk-fed counterparts. Formula alone failed to provide >2 mg/kg/day of iron in 80% of infants.

Average total iron intake in the non-breast-milk-fed group was 2.6 mg/kg/day (combined from formula and supplements), compared with 2 mg/kg/day in the breast-milk-fed group. Based on this, one would expect ID to be comparable or even less prevalent in the non-breast-milk-fed group, however, this was not the case. The prevalence of ID was significantly higher in the non-breast-milk-fed group (36.8%) than in the breast-milk-fed group (20.6%). The non-breast-milk-fed group had lower mean maternal age and higher prevalence of smoking and single parent status. However, even after accounting for the maternal and neonatal differences between two groups, odds of ID remained higher in the non-breast-milk-fed group.

The literature examining the effect of breast-milk-feeding versus feeding with iron-fortified formula on the iron status of preterm infants is scarce [19]. A sole observational study from the Netherlands examined 92 infants fed with iron-fortified formula and 46 infants fed with human milk [20]. Iron supplementation was provided until only 3 months CA in both groups, and ID was tested for at 6 months CA. The incidence of ID was lower in formula-fed infants (9.5%) compared to infants fed human milk (26%). These results are not directly comparable to our study since most breast-milk-fed infants in our study (79%) were still on iron supplements at 4–6-months CA. The infants in this study also had a higher average gestational age (30 weeks) and birthweight (1375 g) compared to those in our study. It is likely that the more premature infants in our study would have had higher iron requirements, and therefore would be less likely to have those requirements met by formula alone. 

When calculated from the formula alone (without supplements), total iron intake in the formula fed infants was >2 mg/kg/day in only 20% infants. This is very important information since it is often thought that the formula has enough iron that easily provides 2 mg/kg/day of iron to a preterm infant. In the 2019 CPS guidelines, authors suggest that iron-rich formulas provide 2–3 mg/kg/day of elemental iron for infants weighing <2 kg at birth and therefore do not recommend additional iron supplementation for these infants [14]. In contrast, our study showed that 80% of formula-fed VPI fail to receive >2 mg/kg/day of iron from formula alone at 4–6 months CA. These results challenge the CPS assumption that formulas provide enough iron for all infants weighing <2 kg at birth. This observation also implies that many formula-fed VPI may require additional iron supplementation outside of infant formula. 

Higher prevalence of ID in the non-breast-milk-fed group, despite higher iron intake, is a result that seems counterintuitive. We hypothesise that this result could be attributed to a difference in iron absorption between breast-milk-fed and non-breast-milk-fed babies [21]. A study comparing the biomolecules to which iron is bound in breast-milk and formula using mass spectrometry found that most iron in commercial formulas is bound to low molecular weight molecules, while most iron in breast-milk is bound to high molecular weight molecules. They concluded that the low molecular weight of iron species in formula decreases the bioavailability of iron [22]. Additionally, the hormone lactoferrin, present in high concentrations in breast-milk, may contribute to increased iron absorption in infants [23,24]. A recent study showed that breast-milk-fed infants have lower levels of serum hepcidin than formula-fed infants, despite similar urinary ferritin levels [25]. Hepcidin is a hormone that has been shown to sequester iron in hepatocytes, diminishing the serum iron available for use in erythropoiesis [26,27]. Higher levels of hepcidin could lead to lower levels of serum iron in formula-fed VPI.

A secondary objective for the study was to identify risk factors for ID in formula-fed VPI. Gestational hypertension was independently associated with ID in these infants. A possible explanation could be increased hepcidin levels in mothers with gestational hypertension. Maternal serum hepcidin levels are low in healthy pregnancies [28]. However, in gestational hypertension, both serum and cord hepcidin levels tend to rise, which may reduce the transfer of iron from mother to fetus and may contribute to ID in the neonate [29]. In a study of 710 children authors found that elevated cord blood hepcidin at birth was associated with increased incidence of anemia in the first four years of life [30]. In contrast, extremely preterm infants have been shown to regulate their own hepcidin levels and iron status [31]. The impact of gestational hypertension on the iron status of VPI, as well as the potential role of hepcidin, needs to be explored further. 

Birthweight <1100 g was also an independent risk factor for ID in formula-fed VPI. This may be due to even lower iron stores in more immature infants, as most iron is accrued from the mother during the last trimester of pregnancy. Since total body iron at birth is approximately 75 mg/kg, we would also expect smaller infants to be born with less iron overall. Additionally, these infants are more likely to experience a variable neonatal course, variable blood transfusions, and undergo variable growth, making it difficult at times to ascertain their exact iron requirements [6,7,8]. This finding highlights the need for a risk-adapted strategy based on birthweight and other clinical factors when deciding iron requirements for VPI. The current CPS, AAP and European guidelines do not distinguish the iron needs of formula-fed preterm infants based on the degree of prematurity [14]. 

ID infants were more likely to have received blood transfusions during their neonatal stay. Although this was not an independent association, it was an important observation. ID may have been higher in this group because of higher phlebotomy losses. Authors also speculate that clinicians may be more likely to withhold iron supplementation for infants who have received blood transfusions out of concern for iron overload. Withholding iron supplementation may not be ideal following a blood transfusion unless there is concern regarding a hemolytic etiology [12,32].

Strengths of this study include its large sample size and population-based study design. One study limitation is unavailable laboratory data for 156 infants. However, adequate blood sampling is often challenging in tiny infants, so this would have been difficult to prevent. Another limitation is the lack of data on other markers of ID, such as transferrin saturation. As ferritin is an acute phase reactant, it may be falsely elevated with infection or inflammation. The infants in this study, though, were not experiencing any illness at the time of follow-up, so ferritin should be an acceptable marker of iron status. The use of additional markers of ID would have likely overestimated the prevalence of ID in the study population but may not have affected the 2 groups differently. The retrospective study design is also a limitation; however, all clinical information was prospectively captured in the database. Another limitation is that researchers were unable to measure iron intake from other sources, such as solid foods. However, at 4–6-months CA, solid foods do not make up a significant proportion of a child’s diet and would not have significantly contributed to total iron intake, Further, this would not have affected the two feeding groups differently. 

## 5. Conclusions

Non-breast-milk-fed VPI had a high prevalence of ID, despite higher iron intake than their breast-milk-fed counterparts. This underlines the need to monitor iron stores in non-breast-milk-fed VPI, and to recommend additional iron supplementation for them. Using a risk-adapted strategy when deciding on iron supplementation to these infants could be helpful [32]. These observations suggest the need to revisit international recommendations for iron supplementation in these infants. Further research on the bioavailability of iron from formula versus from breast-milk to corroborate these findings is important. Addition of more bioavailable iron species to formulas should be considered over increasing the iron content of formulas in order to prevent ID in formula-fed VPI.

## Figures and Tables

**Table 1:** NICU characteristics of infants who were breast-milk-fed vs. non-breast-milk-fed at 4–6-months corrected age (N = 392).

Variables	Non-Breast-Milk-Fed Infants N = 285 *n*(%)	Breast-Milk-Fed Infants N = 107 *n*(%)	Odds Ratio or Mean Difference (95% CI)
Antenatal Variables			
Maternal age, years, mean ± SD *	29.2 ± 5.9	31.9 ± 4.8	2.7 (1.6 to 3.9)
Maternal anemia	20 (7.1)	2 (1.9)	4.0 (0.91, 17.3)
Gestational hypertension	51 (17.9)	16 (14.9)	1.2 (0.67, 2.3)
Smoking *	88 (30.9)	7 (6.5)	6.6 (2.9, 14.7)
Maternal diabetes	17 (6.0)	5 (4.7)	1.3 (0.47, 3.6)
Antepartum hemorrhage	47 (16.5)	20 (18.7)	0.87 (0.49, 1.55)
Multiple gestation *	82 (28.8)	42 (39.3)	0.63 (0.39, 0.99)
Mode of delivery			
Vaginal	116 (40.7)	49 (45.8)	Ref
Cesarean section	169 (59.3)	58 (54.2)	1.2 (0.79, 1.93)
Neonatal Variables			
Gestational age, weeks, mean ± SD	27.8 ± 1.9	28 ± 1.7	0.18 (−0.22 to 0.58)
Gestational age, weeks			
23–27	108 (37.9)	36 (33.6)	1.20 (0.75, 1.92)
28–30	177 (62.1)	71 (66.4)	Ref
Birth weight, grams, mean ± SD	1133 ± 317	1172 ± 318	38.52 (−32 to 109)
Birth weight, grams <1100 ≥1100			
	133 (46.7)	46 (43.0)	1.2 (0.74, 1.82)
	152 (53.3)	61 (57.0)	Ref
Length of hospital stay, days, mean ± SD	78 ± 49	76 ± 34	−2.14 (−10.87 to 6.59)
Need of any blood transfusions	150 (52.6)	61 (57.0)	0.86 (0.55, 1.34)
Hemoglobin at discharge, g/L, mean ± SD	111 ± 25	109 ± 20	−2.67 (−7.62 to 2.29)
Ferritin at discharge, µg/L, mean ± SD	93 ± 92	102 ± 93	8.55 (−17.37 to 34.46)
Feeding at NICU discharge Mixed (Breast and formula) Exclusive Breast-feeding Exclusive Formula-feeding			
	134 (47.0)	95 (88.9)	0.04 (0.01–0.1)
	10 (3.5)	8 (7.5)	0.04 (0.01–01)
	141 (49.5)	4 (3.7)	Ref
Dose of iron at discharge, mg/kg/d, mean ± SD *	3.0 ± 1.1	3.3 ± 1.2	0.28 (0.02 to 0.53)
Male sex	155 (54.4)	63 (58.9)	0.84 (0.54, 1.3)
BPD requiring oxygen at 36 weeks	64 (22.5)	17 (15.9)	1.5 (0.85, 2.8)
HS-PDA	73 (25.6)	24 (22.4)	1.2 (0.70, 2.0)
Necrotizing enterocolitis	9 (3.2)	2 (1.9)	1.7 (0.37, 8.1)
Culture positive sepsis	56 (19.6)	22 (20.6)	0.95 (0.54, 1.64)
Intraventricular hemorrhage, any grade	93 (32.6)	33 (30.8)	1.1 (0.67, 1.75)
Cystic brain injury	14 (4.9)	7 (6.5)	0.74 (0.29, 1.9)
Sociodemographic Variables			
Single parent *	36 (12.6)	4 (3.7)	3.8 (1.3, 11.1)
Urban dweller (vs. rural)	225 (78.9)	89 (83.2)	0.77 (0.43, 1.4)

Note: CI: confidence intervals, SD: standard deviation, BPD: bronchopulmonary dysplasia, HS-PDA: hemodynamically significant patent ductus arteriosus; Ref, reference; vs., versus. Note: * indicates *p* < 0.05.

**Table 2:** Post-discharge characteristics of infants who were breast-milk-fed vs. non-breast-milk-fed at 4–6-months corrected age (N = 392).

Variable	Non-Breast-Milk-Fed Infants N = 285 *n*(%)	Breast-Milk-Fed Infants N = 107 *n*(%)	Odds Ratios/Mean Differences (95% CI)	*p*-Values
Corrected age at time of assessment, months (mean ± SD)	4.9 ± 1.2	4.9 ± 1.1	0.07 (−0.18 to 0.33)	0.58
Formula intake (mL/kg/day) (mean ± SD) *	132± 40	37 ± 44	−95.25 (−104.89 to −85.60)	<0.001
Iron obtained from formula, mg/kg/day, mean ± SD *	1.66 ± 0.54	0.43 ± 0.54	−1.22 (−1.35 to −1.10)	<0.001
Iron supplements *	165 (57.9)	85 (79.4)	0.36 (0.21, 0.60)	<0.001
Iron intake from supplement, mg/kg/day, mean ± SD *	0.93 ± 1.00	1.59 ± 1.09	0.66 (0.42 to 0.89)	<0.001
Total iron intake, mg/kg/day, mean ± SD *	2.59 ± 1.22	2.02 ± 1.23	−0.57 (−0.85 to −0.30)	<0.001
Anti-reflux medication	105 (36.8)	41 (38.3)	0.94 (0.60, 1.49)	0.79
Iron deficiency *	105 (36.8)	22 (20.6)	2.25 (1.33, 3.82)	0.002

Note: * indicates *p* < 0.05. Abbreviations: CI, confidence interval; kg, kilogram; mg, milligram; SD, standard deviation.

**Table 3:** Iron indices in breast-milk-fed and non-breast-milk-fed infants at 4–6-months corrected age (N = 392).

Marker	Non-Breast-Milk-Fed Infants N = 285 mean ± SD	Breast-Milk-Fed Infants N = 107 mean ± SD	Mean Differences (95% CI)	*p*-Values
Ferritin, µg/L *	26.8 ± 18.4	44.8 ± 38.1	17.9 (12.3–23.6)	<0.001
Ferritin, µg/L * median (IQR)	20.4 (17.3)	31.3 (39.4)		<0.001
MCV, fL *	78.8 ± 3.1	77.7 ± 3.8	−1.1 (−1.8 to −0.4)	0.004
MCH, pg *	27.4 ± 1.2	26.9 ± 1.6	−0.6 (−0.8 to −0.3)	<0.001
MCHC, g/L *	348.4 ± 8.5	346.1 ± 10.6	−2.3 (−4.4 to −0.3)	0.02
RDW, %	12.6 ± 1.3	12.9 ± 0.8	0.2 (0.0–0.5)	0.07
RetCount, %	1.1 ± 0.3	1.1 ± 0.3	0.0 (−0.1 to 0.0)	0.24
Ret-He, pg	30.4 ± 2.1	30.5 + 3.2	0.1 (−0.8 to 0.9)	0.91
Hemoglobin, g/L	124.1 ± 9.3	122.3 ± 10.7	−1.8 (−4.0 to 0.4)	0.10

Note: * indicates *p* < 0.05. Abbreviations: CI, confidence interval; fL, femtoliters; g/L, grams per litre; pg, picograms; SD, standard deviation; µg/L, micrograms per litre; IQR, interquartile range.

**Table 4:** NICU characteristics of exclusively formula-fed preterm infants who were iron deficient vs. not iron deficient at 4–6-months corrected age (n = 285).

Variables	ID N = 105 *n* (%)	Non-ID N = 180 *n* (%)	OR or Mean Difference (95% CI)
Antenatal Variables			
Maternal age, years, mean ± SD	30.1 ± 6.2	28.7 ± 5.8	−1.43 (−2.9 to 0.03)
Maternal anemia	7 (6.7)	13 (7.2)	0.92 (0.35, 2.37)
Gestational hypertension *	27 (25.7)	24 (13.3)	2.25 (1.22, 4.16)
Smoking	31 (29.5)	57 (31.7)	0.93 (0.55, 1.58)
Maternal diabetes	6 (5.7)	11 (6.1)	0.93 (0.33, 2.59)
Antepartum hemorrhage	19 (18.1)	28 (15.6)	1.20 (0.63, 2.27)
Multiple gestation	30 (28.6)	52 (28.9)	1.00 (0.59, 1.70)
Mode of delivery *			
Vaginal	34 (32.4)	82 (45.6)	Ref
Cesarean section	71 (67.6)	98 (54.4)	1.75 (1.06, 2.89)
Neonatal Variables			
Gestational age, weeks, mean ± SD *	27.5 ± 2.0	28.0 ± 1.9	0.52 (0.05 to 0.99)
Gestational age, weeks			
23–27	45 (42.9)	63 (35.0)	1.39 (0.85, 2.28)
28–30	60 (57.1)	117 (65.0)	Ref
Birth weight, grams, mean ± SD *	1069 ± 294	1170 ± 326	101.09 (27.01 to 175.17)
Birth weight, grams *			
<1100	59 (56.2)	74 (41.1)	1.84 (1.13, 2.99)
≥1100	46 (43.8)	106 (58.9)	Ref
Length of hospital stay, days, mean ± SD	80 ± 56	77 ± 46	−2.89 (−15.89 to 10.12)
Need of any blood transfusions during neonatal stay *	65 (61.9)	85 (47.2)	1.76 (1.08, 2.88)
Hemoglobin at discharge, g/L, mean ± SD	109 ± 20	113 ± 28	3.94 (−1.73 to 9.60)
Ferritin at discharge, µg/L, mean ± SD	85 ± 81	99 ± 98	13.97 (−12.44 to 40.37)
Dose of iron at discharge, mg/kg/d, mean ± SD	3.0 ± 1.1	3.0 ± 1.1	0.00 (−0.26 to 0.27)
Male sex	63 (60.0)	92 (51.1)	1.47 (0.90, 2.40)
BPD requiring oxygen at 36 weeks	28 (26.7)	36 (20.0)	1.48 (0.84, 2.62)
HS-PDA	31 (29.5)	42 (23.3)	1.38 (0.80, 2.37)
Necrotizing enterocolitis	3 (2.9)	6 (3.3)	0.86 (0.21, 3.52)
Culture positive sepsis	26 (24.8)	30 (16.7)	1.65 (0.91, 2.97)
Intraventricular hemorrhage, any grade	32 (30.5)	61 (33.9)	0.86 (0.51, 1.44)
Cystic brain injury	8 (7.6)	6 (3.3)	2.42 (0.82, 7.17)
Sociodemographic Variables			
Single parent	15 (14.3)	21 (11.7)	1.31 (0.64, 2.67)
Urban dweller (vs. rural)	81 (77.1)	144 (80.0)	0.88 (0.49, 1.59)

Note: * indicates *p* < 0.05. OR: odds ratio, CI: confidence intervals, SD: standard deviation, BPD: bronchopulmonary dysplasia, HS-PDA: hemodynamically significant patent ductus arteriosus; Ref, reference.

**Table 5:** Post-discharge characteristics of exclusively formula-fed preterm infants who were iron deficient vs. not iron deficient at 4–6-months corrected age (N = 285).

Variable	ID N = 105 *n* (%)	Non-ID N = 180 *n* (%)	Odds Ratios and Mean Differences (95% CI)	*p*-Values
Corrected age at time of assessment, months (mean ± SD) *	4.4 ± 0.88	5.2 ± 1.20	0.78 (0.53 to 1.02)	<0.001
Post-discharge Preterm formula *	59 (56.2)	45 (25.0)	3.85 (2.31–6.42)	<0.001
Formula intake, mL/kg/day (mean ± SD) *	142 ± 43	127 ± 36	−14.75 (−24.62 to −4.87)	0.004
Iron obtained from formula, mg/kg/day, mean ± SD *	1.83 ± 0.57	1.55 ± 0.49	−0.27 (−0.40 to −0.14)	<0.001
Iron supplements	66 (62.9)	99 (55.0)	1.39 (0.85–2.27)	0.20
Iron intake from supplement, mg/kg/day, mean ± SD	1.08 ± 1.05	0.85 ± 0.95	−0.24 (−0.48 to 0.01)	0.06
Total iron intake, mg/kg/day, mean ± SD *	2.92 ± 1.27	2.40 ± 1.16	−0.52 (−0.82 to −0.22)	<0.001
Anti-reflux medication	44 (41.9)	61 (33.9)	1.41 (0.86–2.31)	0.18

Note: * indicates *p* < 0.05. Abbreviations: CI, confidence interval; ID, iron deficient; kg, kilogram; mg, milligram; SD, standard deviation.

**Table 6:** Post-discharge iron indices in iron deficient and non-iron deficient exclusively formula-fed infants at 4–6-months corrected age.

Marker	Iron Deficient N = 105 Mean ± SD	Non-Iron Deficient N = 180 Mean ± SD	Mean Differences (95% CI)	*p*-Values
Ferritin, µg/L *	15.7 ± 5.9	33.2 ± 20.0	17.5 (13.5–21.5)	<0.001
Ferritin, µg/L * median (IQR)	15.4 (6.7)	26.4 (18.8)		<0.001
MCV, fL	78.8 ± 3.1	78.7 ± 3.1	0.0 (−0.8–0.7)	0.92
MCH, pg	27.4 ± 1.2	27.4 ± 1.2	0.0 (−0.3–0.3)	0.81
MCHC, g/L	348.0 ± 8.3	348.6 ± 8.6	0.6 (−1.4–2.7)	0.56
RDW, %	12.5 ± 1.9	12.7 ± 0.7	0.1 (−0.2–0.5)	0.37
RetCount, %	1.1 ± 0.3	1.1 ± 0.3	0.0 (−0.1–0.1)	0.72
Ret-He, pg *	29.7 ± 2.0	30.7 ± 2.0	1.0 (0.2–1.9)	0.02
Hemoglobin, g/L	124.2 ± 9.3	124.1 ± 9.2	−0.1 (−2.3–2.2)	0.93

Note: * indicates *p* < 0.05. Abbreviations: CI, confidence interval; fL, femtoliters; g/L, grams per litre; pg, picograms; SD, standard deviation; µg/L, micrograms per litre.

## Data Availability

The data presented in this study are available on request from the corresponding author. The data are not publicly available due to patient privacy concerns.
